# Full phenology cycle carbon flux dynamics and driving mechanism of Moso bamboo forest

**DOI:** 10.3389/fpls.2024.1359265

**Published:** 2024-02-26

**Authors:** Cenheng Xu, Fangjie Mao, Huaqiang Du, Xuejian Li, Jiaqian Sun, Fengfeng Ye, Zhaodong Zheng, Xianfeng Teng, Ningxin Yang

**Affiliations:** ^1^State Key Laboratory of Subtropical Silviculture, Zhejiang Agriculture and Forestry University, Lin’an, Zhejiang, China; ^2^Key Laboratory of Carbon Cycling in Forest Ecosystems and Carbon Sequestration of Zhejiang Province, Zhejiang Agriculture and Forestry University, Lin’an, Zhejiang, China; ^3^School of Environmental and Resources Science, Zhejiang Agriculture and Forestry University, Lin’an, Zhejiang, China

**Keywords:** Moso bamboo forest, full phenology cycle, carbon flux, photosynthetic parameters, driving force analysis

## Abstract

**Introduction:**

Moso bamboo forests, widely distributed in subtropical regions, are increasingly valued for their strong carbon sequestration capacity. However, the carbon flux variations and the driving mechanisms of Moso bamboo forest ecosystems of each phenology period have not been adequately explained.

**Methods:**

Hence, this study utilizes comprehensive observational data from a Moso bamboo forest eddy covariance observation for the full phenological cycle (2011-2015), fitting a light response equation to elucidate the evolving dynamics of carbon fluxes and photosynthetic characteristics throughout the entire phenological cycle, and employing correlation and path analysis to reveal the response mechanisms of carbon fluxes to both biotic and abiotic factors.

**Results:**

The results showed that, First, the net ecosystem exchange (NEE) of Moso bamboo forest exhibits significant variations across six phenological periods, with LS_OFF_ demonstrating the highest NEE at -23.85 ± 12.61 gC·m^-2^·5day^-1^, followed by LS_ON_ at -19.04 ± 11.77 gC·m^-2^·5day^-1^ and FG_ON_ at -17.30 ± 9.58 gC·m^-2^·5day^-1^, while NF_OFF_ have the lowest value with 3.37 ± 8.24 gC·m^-2^·5day^-1^. Second, the maximum net photosynthetic rate (P_max_) and apparent quantum efficiency (α) fluctuated from 0.42 ± 0.20 (FG_ON_) to 0.75 ± 0.24 mg·m^-2^·s^-1^ (NF_OFF_) and from 2.3 ± 1.3 (NF_OFF_) to 3.3 ± 1.8 μg·μmol^-1^ (LS_OFF_), respectively. Third, based on the path analysis, soil temperature was the most important driving factor of photosynthetic rate and NEE variation, with path coefficient 0.81 and 0.55, respectively, followed by leaf area index (LAI), air temperature, and vapor pressure difference, and precipitation. Finally, interannually, increased LAI demonstrated the potential to enhance the carbon sequestration capability of Moso bamboo forests, particularly in off-years, with the highest correlation coefficient with NEE (-0.59) among the six factors.

**Discussion:**

The results provide a scientific basis for carbon sink assessment of Moso bamboo forests and provide a reference for developing Moso bamboo forest management strategies.

## 1 Introduction

Forest carbon flux is a major component of the terrestrial ecosystem carbon cycle, accounting for over 90% of the total carbon exchanged between terrestrial ecosystems and the atmosphere (Friedlingstein et al., 2022), and plays an important role in maintaining regional ecological balance (Iturbide et al., 2020; Zhao et al., 2022). Forest carbon flux monitoring methods mainly include sample plot inventory, model simulation, and micrometeorological methods (Zhao et al., 2022). The sample inventory method is the most basic and accurate, but it comprises a large workload and is easily constrained by time and space. Model simulation includes statistical, parameter, and process-based models, which can provide support for studying forest ecosystem carbon cycling on a large scale, but due to spatiotemporal complexities and input parameter uncertainty, the simulation results of different models differ considerably (Mao et al., 2017a). The micrometeorological method usually refers to the eddy covariance CO_2_ flux observation technique, the only method for directly determining the exchange of community CO_2_ with the atmosphere that is widely used in global carbon flux observations (Gong et al., 2020). The flux observation networks, such as AmeriFlux, ChinaFlux, AsiaFlux and FLUXNET, provide important data for observing ecological phenomena from individual and community levels to the dynamic changes in ecosystem functions on a large scale. For example, Harris et al. (2021) used FLUXNET observations to map global forest carbon flux in the 21st century; Chu et al. (2021) evaluated flux footprints and the representativeness of these footprints for target areas by AmeriFlux; Chang et al. (2023) combined random forest with ChinaFlux data to estimate the GPP for the 9 sites. Therefore, the use of eddy covariance system to monitor the dynamics of regional carbon fluxes is an effective and currently well-respected approach.

Biotic and abiotic factors are important factors affecting carbon fluxes in forest ecosystems, and the extent and mechanisms of their effects are complicated by different vegetation physiological characteristics and growing environments (Baldocchi et al., 2018). For example, Xie et al. (2014) found that an increase in temperature reduces carbon sequestration by increasing respiration, but Richardson et al. (2010) pointed out that warming in a certain range increases photosynthesis, which increases ecosystem carbon sequestration. Regardless, it has become a scholarly consensus that ecosystem respiration, a major factor in carbon emissions, is primarily influenced by temperature, especially in moisture-rich regions (Kondo et al., 2017). However, besides abiotic factors such as temperature and radiation, vegetation photosynthesis is also influenced by growth cycles (phenology) and canopy structure (e.g., leaf area index [LAI]) (Gitelson et al., 2017). Therefore, analyzing the characteristics of photosynthetic carbon fixation and elucidating the effect of photosynthesis on carbon fluxes is a hotspot in studying the mechanism of carbon fluxes influence in forest ecosystems. Fitting the light response equations of different vegetation is an important method for understanding dynamic plant physiology processes (Zhang et al., 2006; Li et al., 2014; Zhou et al., 2017). Apparent quantum efficiency (*α*) and maximum photosynthetic rate (P_max_) are important characteristic parameters in the vegetation light response equation, which can accurately describe the characteristics of vegetation photosynthesis and its intensity (Lin et al., 2022).

Subtropical forest ecosystems in the East Asian monsoon zone has a non-negligible role in mitigating global warming, and its net ecosystem productivity is 0.72 Pg C·a^-1^ (Yu et al., 2014). Moso bamboo (*Phyllostachys edulis*) is a special forest type widely distributed in subtropical areas, with an annual NEE approximately of -105.2 gC·m^-2^·a^-1^, indicating a strong pathway model carbon sequestration potential for mitigating climate change (Song et al., 2020). However, Moso bamboo has special phenological and growth characteristics, i.e., the alternation of on- and off-years (mass of bamboo shoots in one year, and almost none in another), and “explosive growth” of new bamboo (Mao et al., 2017a). Several studies explored the spatiotemporal patterns of carbon storage, productivity and carbon fluxes and their response to climate change (Mao et al., 2022; Yan et al., 2023), such as Mao et al. (Mao et al., 2016; Mao et al., 2017a) adapted the BIOME-BGC model for the simulation of managed Moso bamboo forest ecosystems, and simulated the carbon fluxes of bamboo forests in Zhejiang Province, China (Mao et al., 2017b); Kang et al. (2022) used the BEPS model to simulate the carbon fluxes of bamboo forests in China; Li et al. (2021) estimated GPP of subtropical bamboo forests by assimilated-LAI. In addition, the start and length of the growing season of subtropical bamboo forest had been successfully retrieved using LAI and SIF datasets (Li et al., 2023; Xu et al., 2023). However, the key drivers under different time scalars are still unclear, such as Liu et al. (2018) found the most important factor affecting net ecosystem change (NEE) and respiration (RE) at daily scalar was vapor pressure difference (VPD), while at monthly scalar was soil temperature (Ts). Zhou et al. (2019) indicated that the effect of biotic and abiotic factors differs in on- and off-years. Moreover, lacks of the carbon flux dynamics and driving mechanism throughout the whole phenology cycle of Moso bamboo forests, bring huge uncertainties in accurately assessing the response of bamboo forests to climate change at a large spatial scale (Huang et al., 2023; Xu et al., 2023; Yan et al., 2023).

Therefore, this study obtained and correlated the carbon fluxes, biotic and abiotic factors of full phenology cycle of Moso bamboo forests based on the eddy covariance observation from 2011 to 2015, analyzed the dynamic and differences of carbon fluxes and photosynthetic indices during six Moso bamboo specific phenology period, and finally quantitatively analyzed the direct and indirect effects of abiotic and biotic factors on carbon fluxes using the combination of correlation and path analysis methods.

## 2 Materials and methods

### 2.1 Study area

**Figure 1:**
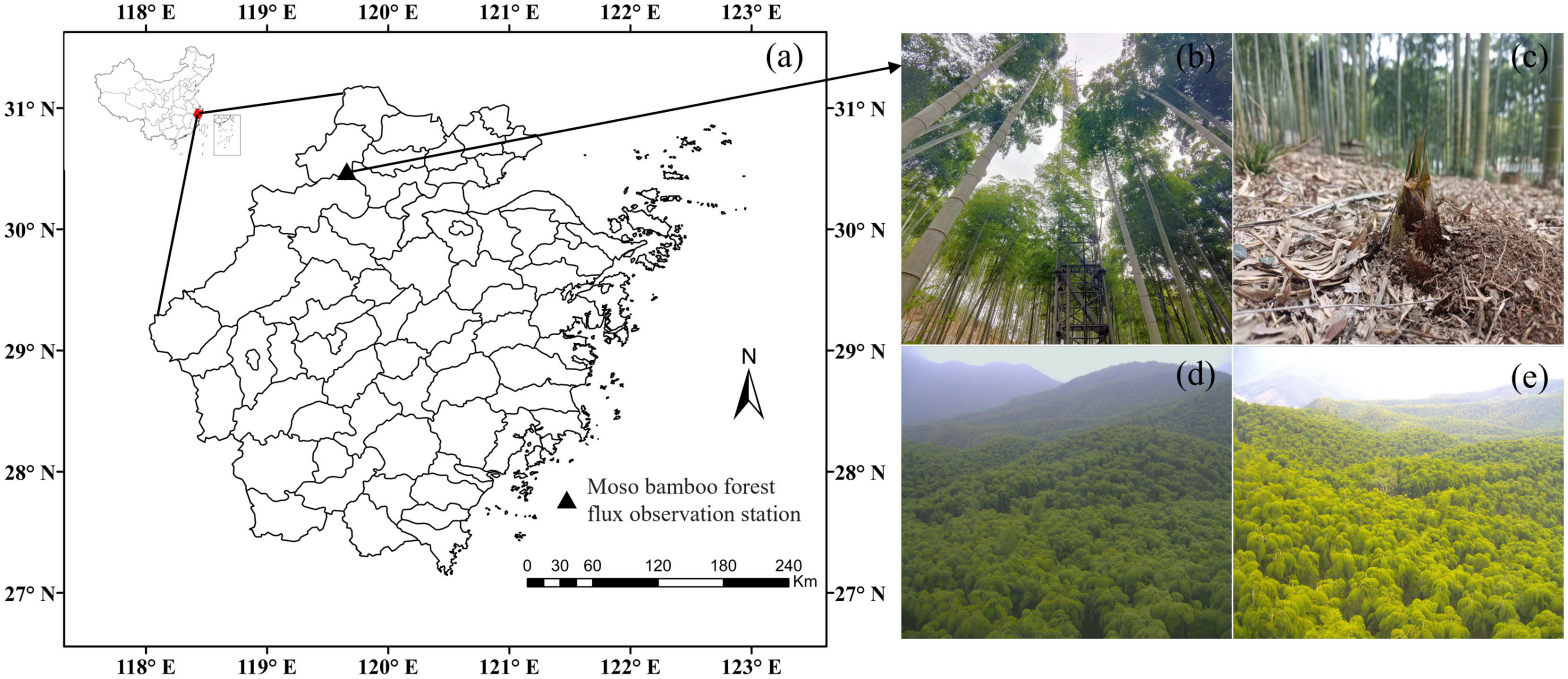
Location of the Moso bamboo forest ecosystem carbon flux observation site **(A)** as well as images of the tower **(B)**, bamboo shoots **(C)**, and spring **(D)** and autumn canopies **(E)** of the Moso bamboo forest.

The Moso bamboo forest ecosystem flux observation station(**Figures 1A, B**) is located in Anji County, Zhejiang Province, China (30.46°N, 119.66°E). The forest area is 13.8 × 10^4^ hm^2^, the forest coverage rate is 71% in Anji. The climate type is subtropical monsoon, the average annual temperature is 16.6 °C, the average annual precipitation is 1400 mm, and the annual sunshine hours are 2021 h. The altitude of the flux observation station is 380 m, the terrain is flat in the southeastern and southern parts of the observation tower, and the slope in the northwestern and northern parts of the observation tower ranges from 2.5° to 14°, and the Moso bamboo forest is dominant within 1 km × 1 km of the observation tower. The area of Moso bamboo forest is 2155 hm^2^, with an average crown height of 11 m, an average diameter at breast height of 9.3 cm, and yellow loam and yellow-red loam soil types, with sparse herbs and shrubs in the understory, which are pure Moso bamboo forests operated by artificial rough management (Liu et al., 2018). The growth cycle of Moso bamboo forests comprises a 2-year cycle, with a first year, known as on-year, comprising a large number of shoots, and a second year, known as off-year, comprising a small number of shoots, in which the growth period is generally concentrated in March–September (Li et al., 2019).

### 2.2 Sample survey

#### 2.2.1 Carbon fluxes and micrometeorological data in Moso bamboo forest ecosystems

Carbon fluxes and micrometeorological data were obtained by 40 m high flux tower equipped with open-path eddy-covariance system (OPEC), atmospheric profile system (APS), and gradient micrometeorological system (GMS). The OPEC was deployed at 38 m according to the height of the forest stand canopy, while the APS and GMS were deployed at seven levels (1, 7, 11, 17, 23, 30, and 38 m) on both sides of the tower arm. The OPEC comprised a 3-D sonic anemometer (CSAT3, Campbell Scientific, USA) and an open-path CO_2_/H_2_O analyzer (Li-7500, Li-COR Biosciences, USA). The APS was deployed to obtain real-time CO_2_ and H_2_O concentration by AP200(Campbell Scientific, USA). The GMS included temperature and humidity sensor (HMP155, Vaisala, Finland), wind speed sensor (WindSonic, Gill Instruments, UK), 4-component net radiometer (CNR4, Campbell Scientific, USA), soil moisture and temperature profile sensor (SoilVUE10, Campbell Scientific, USA) and soil heat flux sensor (HFP01, Hukseflux, Netherlands) at depths of 5, 10, 20, 30, 40 and 50 cm. Systematic observation data, including physical quantities such as CO_2_ flux at 10 Hz, friction wind speed, and other relevant physical quantities, as well as 30-min averaged conventional meteorological information, were stored using a CR1000 data collector (Campbell Scientific, USA).

#### 2.2.2 LAI data

LAI was based on sample LAI combined with MODIS LAI and reflectance to assimilate an LAI time series. The sample LAI was determined using a WinSCANOPY canopy analysis system (Regent Instruments, Canada). The specific method included setting up five fixed sample points within 1000 m from the flux tower as the center. To ensure LAI measurement accuracy and avoid light spot formation on the image due to solar radiation, measurement was conducted at 6:00–10:00 and 15:00–17:50 monthly, when it was sunny, without cumulus clouds, and with good atmosphere visibility. Canopy images were obtained using the fisheye lens that came with the canopy analyzer, brought back to the laboratory, and post-processed using the corresponding software. The average of five sample points was taken as the LAI measurement in the field.

The MODIS LAI data assimilation system mainly used the particle filter assimilation algorithm to assimilate MODIS LAI, reflectance data, and PROSAIL model-simulated canopy reflectance into the LAI dynamic model to obtain a high-precision bamboo forest LAI time series product (Fang et al., 2003; Li et al., 2017).

#### 2.2.3 Observation of Moso bamboo phenology

For the purpose of economic beneficial, the farmers usually harvested nearly all Moso bamboo of six years or older in the autumn, leading to a completely renewal of the bamboo stand every five years. This renewal cycle forms the basis of the full phenological cycle studied here. The Moso bamboo phenological observation including bamboo shooting, explosive growth, leaf spreading, and leaf renewing (**Figures 1C-E**) during 2011 - 2015 using a camera deployed on 25 m of the tower, and the lens faces south with an inclination of 20° (Xu et al., 2023). In this study, 2011, 2013 and 2015 belong to on-years, and others were off-years. Combined the phenological characteristics and on- (off-) year phenomena, the full phenology cycle was divided into six periods, and determined the start and end dates of each phenological period by phenology camera observations. The six periods are as follows: (1) fast-growing period (FG_ON_), which refers to the stage when freshly sprouted culms grow above the ground and accomplish their height growth; (2) leaf-spreading in on-years (LS_ON_), indicating the stage when freshly sprouted bamboo culms start flushing leaves; (3) leaf-renewing period (LR_OFF_), describing the stage odd-year-old established culms shed old leaves and flush new leaves; (4) leaf-spreading in off-years (LS_OFF_), which pertains to the stage new leaves are expanding on the odd-year-old established culms; (5, 6) other normal days in on- and off-years (NF_ON_ and NF_OFF_). For the details of each phenology period, please refer to Mei et al. (2020).

### 2.3 Data processing

#### 2.3.1 Half-hourly and daily carbon flux data acquisition

Carbon flux observation was made at the stand canopy level, raw data were processed to daily NEE, RE, and gross ecosystem productivity (GEP) (Mao et al., 2017a). Raw flux data were corrected using EddyPro *v.*6.0.0(LI-COR Inc., USA) by spike removal, tilt correction (double-axis rotation), spectral correction, block averaging, correction for density fluctuations, and subsequent flux calculation (Baldocchi et al., 2018). When atmospheric turbulence is insufficient at night, soil and plant respiration are deposited below the forest canopy, which can easily lead to nighttime flux underestimation; therefore, a friction wind speed rejection threshold of 0.2 m·s^-1^ (Xu et al., 2016a) was adopted in this study.

The steps for missing data interpolation were as follows: first, meteorological data with missing time ≤ 2 h and > 2 h were interpolated using linear interpolation and mean daily variation methods, respectively (Falge et al., 2001); second, the Lloyd–Taylor equation was used to fit the missing RE, by the way, since there is no photosynthesis at night so RE = NEE at night (Lloyd and Taylor, 1994); lastly, the right-angled hyperbolic equation was interpolated to the daytime NEE (Falge et al., 2001) to obtain the complete half-hourly carbon flux time series. On this basis, daily scale carbon fluxes were obtained by accumulation, and GEP calculated by RE minus NEE. In this study negative value of carbon fluxes indicate carbon sink, while positive refers to carbon source.

#### 2.3.2 Extraction of photosynthetic parameters

The daytime 30-min flux samples were too small to fit photosynthetic parameters on a daily scale. Therefore, the apparent quantum efficiency (*α*, mg·μmol^-1^·s^-1^) and maximum photosynthetic rate (P_max_, mg·m^-2^·s^-1^) were fitted using daytime NEE, RE, and PAR data in a 5-day window using the right-angle hyperbolic equation (Equation 1) (Falge et al., 2001). Meanwhile, due to the high frequency of noise fluctuation in the fitting results, *α* and P_max_ were smoothed by Gaussian filter to show the trend of changes (Savitzky and Golay, 1964) of 73 values per year, matching the time series of the flux data.


(1) $$-NEE=\frac{\alpha \times PAR\times P_{max}}{\alpha \times PAR+P_{max}}-RE$$

### 2.4 Data analysis

Correlation and path analysis methods were used to analyze the influence mechanisms of biotic and abiotic factors on carbon fluxes and photosynthetic parameters in a full phenological cycle of Moso bamboo forests.

Based on the above data, the time series of carbon fluxes, photosynthetic parameters, and abiotic factors in different phenological periods of Moso bamboo forest ecosystems from 2011 to 2015 were obtained. Subsequently, the correlation among indicators in different phenological periods was evaluated by using Pearson’s correlation coefficient (*r_xy_*), after which the path coefficient (PC) among indicators was calculated using the pathway model to analyze the direct and indirect impacts of the indicators on carbon fluxes and reveal the degree of influence of each factor on carbon fluxes, and then derive the changes in and driving mechanisms of carbon fluxes and photosynthetic parameters. *r_xy_* was calculated using Equation 2:


(2) $$r_{xy}=\frac{\sum_{i=1}^{n}\left(x_{i}-\bar{x}\right)\left(y_{i}-\bar{y}\right)}{\sqrt{\sum_{i=1}^{n}{\left(x_{i}-\bar{x}\right)}^{2}\sum_{i=1}^{n}{\left(y_{i}-\bar{y}\right)}^{2}}}$$

where, $x_{i}$ is the value of the six biotic and abiotic factors on day i , $y_{i}$ represents the three carbon flux values as well as the two photosynthetic parameters, $\bar{x}$ and $\bar{y}$ represent the total mean values of the biotic and abiotic factors with respect to carbon fluxes and photosynthetic parameters, respectively, n is the total number of days, and i denotes ordinal days (i=1,2,.,n) .

**Figure 2:**
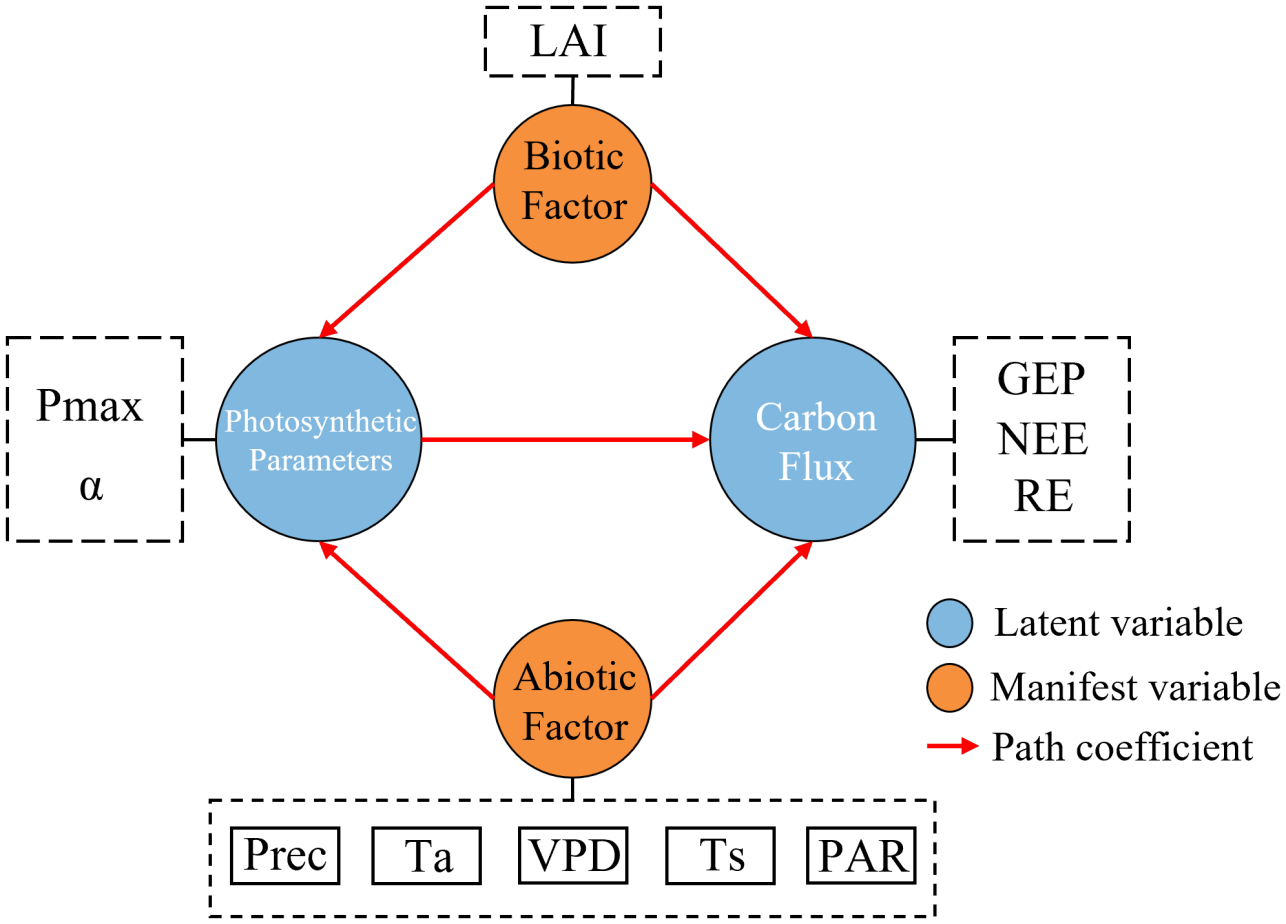
Structure of the inter-variable pathway. VPD, vapor pressure difference; Prec, precipitation; PAR, photosynthetic radiation; LAI, leaf area index; Ta, air temperature; Ts, soil temperature.

Path analysis was conducted using SPSSPRO Ver.1.0.11 (https://www.spsspro.com). NEE was determined using GEP and RE; GEP is directly affected by the photosynthesis (Chen et al., 2009), while P_max_ is important in determining the photosynthetic capacity of ecosystems (Zhang et al., 2006; Flexas and Carriquí, 2020). Based on this logic, the structure of the pathway model constructed in this study is shown in **Figure 2**.

## 3 Results

### 3.1 Characteristics of biotic and abiotic factor changes in Moso bamboo forests

**Figure 3:**
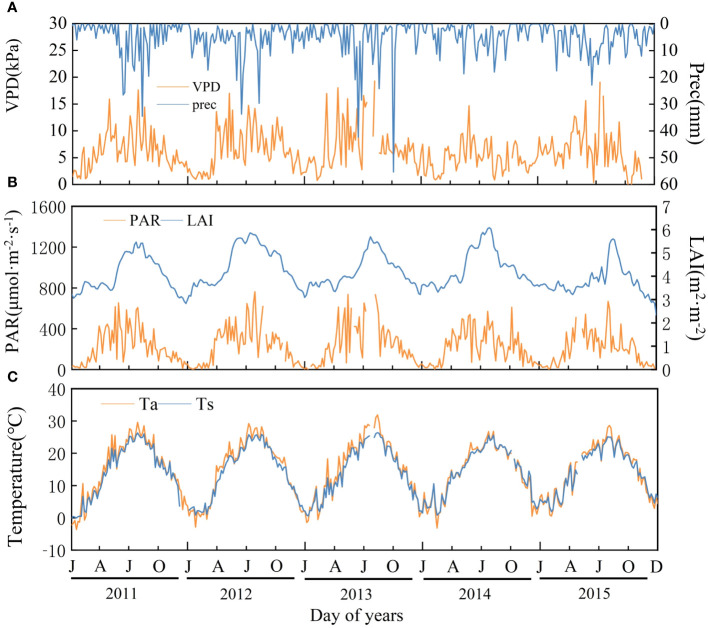
Variation in biotic and abiotic factors of Moso bamboo forests during 2011-2015. **(A)** VPD (vapor pressure difference) and Prec (precipitation); **(B)** PAR (photosynthetic radiation) and LAI (leaf area index); **(C)** Ta, (air temperature) and Ts (soil temperature). Interruptions are missing data.

As shown in **Figure 3**, VPD, precipitation (Prec), PAR, LAI, Ta, and Ts had significant seasonal characteristics, with higher values in summer. VPD fluctuated more, especially in 2013 and 2015, the difference between their maximum and minimum values are 28.93 kPa and 23.11 kPa, respectively, and relatively less in 2011(16.55 kPa), 2012(16.02 kPa) and 2014(13.82 kPa).

The highest annual mean temperature was 15.3°C in 2013, the lowest was 14.1°C in 2014, the highest annual precipitation was 2143 mm in 2012, and the lowest was 1363 mm in 2014. PAR annual average was highest in 2013(260.50 μmol·m^-2^·s^-1^) and lowest in 2015(197.70 μmol·m^-2^·s^-1^). The annual mean values of LAI were 4.02, 4.46, 4.17, 4.34, and 3.78 from 2011 to 2015, respectively. The mean LAI was higher in the off-years (4.40) than that in the on-years (3.99), and the interannual maximum LAI was in summer (2011-2013) or autumn (2014-2015).

### 3.2 Comparison of carbon fluxes and photosynthetic parameters across the full phenological cycle

**Figure 4:**
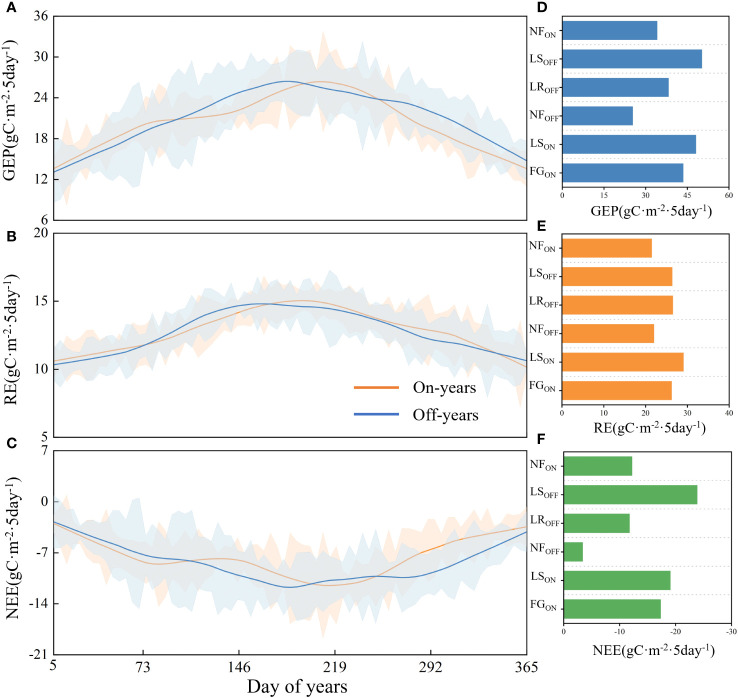
Carbon fluxes in on- and off-years in Moso bamboo forests **(A–C)** and corresponding statistic by every phenological period **(D–F)**. Solid lines are filtered trend lines. Blue shading is standard deviation of off-years, orange is of on-years.

As shown in **Figures 4A–C**, NEE and GEP showed a bimodal pattern in the on-years, with the two peaks occurring in FG_ON_ and LS_ON_, respectively, and in the same period in the off-years, they occurred in LR_OFF_ and LS_OFF_, respectively. The changes in RE exhibited similar trends between on- and off-years. GEP averaged 2871.40 gC·m^-2^·a^-1^in the on-years, with an average maximum of 82.13 gC·m^-2^ in summer and an average minimum of 9.87 gC·m^-2^ in winter, while in the off-years it averaged 2829.49 gC·m^-2^·a^-1^, with an average maximum of 75.08 gC·m^-2^ in summer and an average minimum of 6.17 gC·m^-2^ in winter. NEE averaged -1071.99 gC·m^-2^·a^-1^ in the on-years, with a mean maximum of 11.08 gC·m^-2^ and a minimum of -42.46 gC·m^-2^, and -1051.70 gC·m^-2^·a^-1^ in the off-years, with a mean maximum of 16.99 gC·m^-2^ and a mean minimum of -47.81 gC·m^-2^. The RE in the on-years averaged 1799.41 gC·m^-2^·a^-1^, with a mean maximum of 41.68 gC·m^-2^ and a mean low point of 10.29 gC·m^-2^, and the off-years averaged 1777.79 gC·m^-2^·a^-1^, with a mean maximum of 39.99 gC·m^-2^ and minimum of 4.64 gC·m^-2^.

As shown in **Figures 4D–F**, the mean values of carbon fluxes in six different phenological periods of the on- and off-years were determined according to the on- and off-year time series, starting from FG_ON_ to NF_ON_ as a growth cycle.The mean value of NEE in FG_ON_ was -17.30 ± 9.58 gC·m^-2^, and that in LR_OFF_ was -11.75 ± 12.77 gC·m^-2^; the absolute value of NEE in FG_ON_ was higher than that of LR_OFF_. The mean value of NEE in LS_ON_ was -19.04 ± 11.77 gC·m^-2^, lower than that of NEE in LS_OFF_ at 23.85 ± 12.61 gC·m^-2^. NF_OFF_ had an NEE of -3.37 ± 8.24 gC·m^-2^, while NF_ON_ had an NEE of -12.19 ± 11.42 gC·m^-2^. RE was highest for LS_ON_ at 29.08 ± 4.90 gC·m^-2^; similar for FG_ON_, LR_OFF_, and LS_OFF_ at 26.24 ± 4.04, 26.53 ± 6.32, and 26.39 ± 6.55 gC·m^-2^, respectively, and lower for NF_OFF_ and NF_ON_ at 22.01 ± 4.45 and 21.48 ± 4.82 gC·m^-2^, respectively. GEP was highest in the leaf spreading period, with LS_ON_ and LS_OFF_ at 48.12 ± 12.84 and 50.24 ± 16.15 gC·m^-2^, respectively. FG_ON_ had greater GEP than that of LR_OFF_ (43.54 ± 8.65 vs. 38.28 ± 13.24 gC·m^-2^), whereas NF_OFF_ had the lowest at 25.38 ± 8.56 gC·m^-2^, and NF_ON_ had a slightly higher GEP than that of the NF_OFF_ at 34.12 ± 12.36 gC·m^-2^.

**Figure 5:**
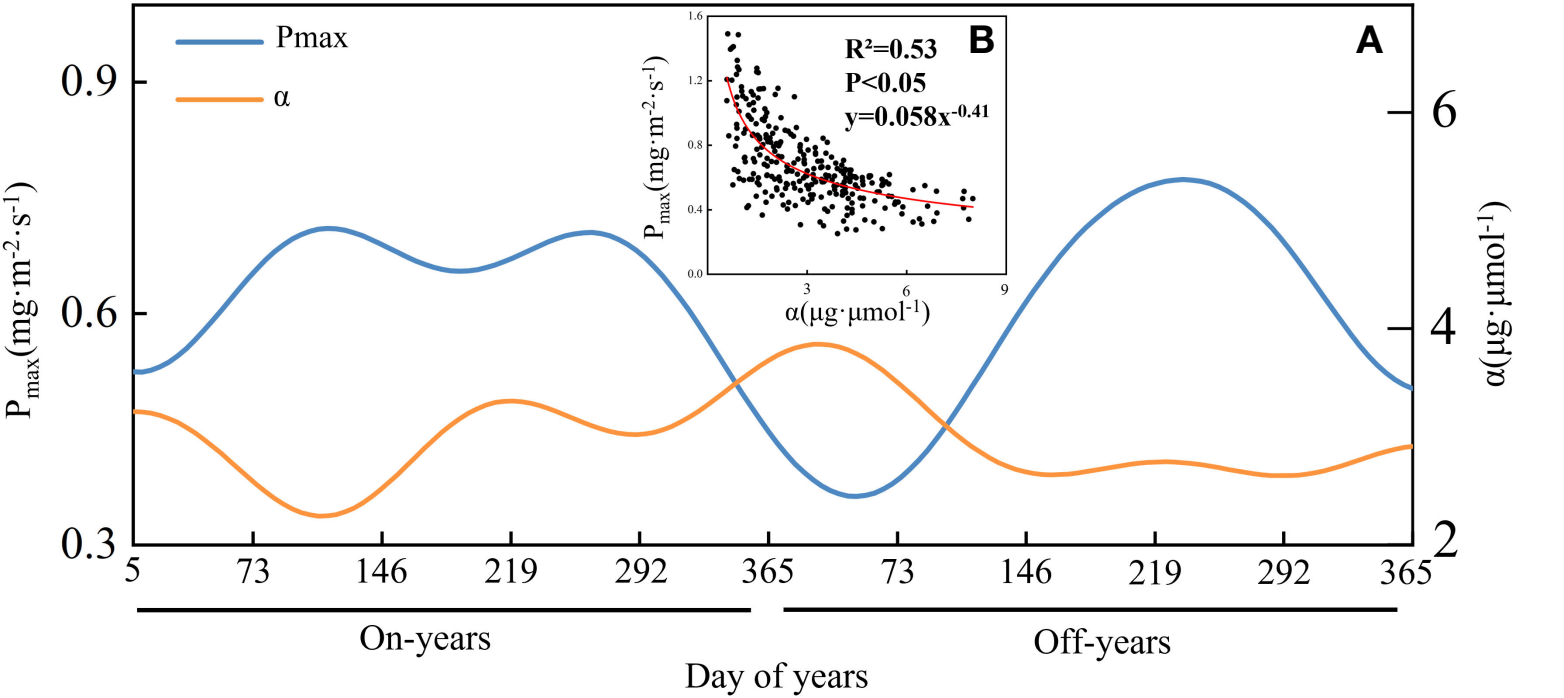
The time series of P_max_ and *α* in Moso bamboo forests **(A)**, as well as their scatterplots **(B)** and allometric fitting curve (red).

As shown in **Figure 5B**, P_max_ and *α* showed a significant negative correlation (*P*< 0.05), and the trend of *α* in an operating cycle was roughly opposite to that of P_max_. As shown in **Figure 5A**, the mean P_max_ value in the on-years (2011, 2013, and 2015) was 0.64 mg·m^-2^·s^-1^ and showed a bimodal pattern of change, with a mean value of *α* of 2.95 μg·μmol^-1^. The mean P_max_ value in the off-years (2012 and 2014) was 0.58 mg·m^-2^·s^-1^ and exhibited a single-peak pattern of change, with a mean *α* of 2.90 μg·μmol^-1^. The P_max_ peak in 2013 was substantially lower than that in 2011 and 2015. The first P_max_ peak in the on-years occurred at FG_ON_, and the second occurred at the end of LS_ON_; the P_max_ peak in off-years occurred at LS_OFF_.

**Figure 6:**
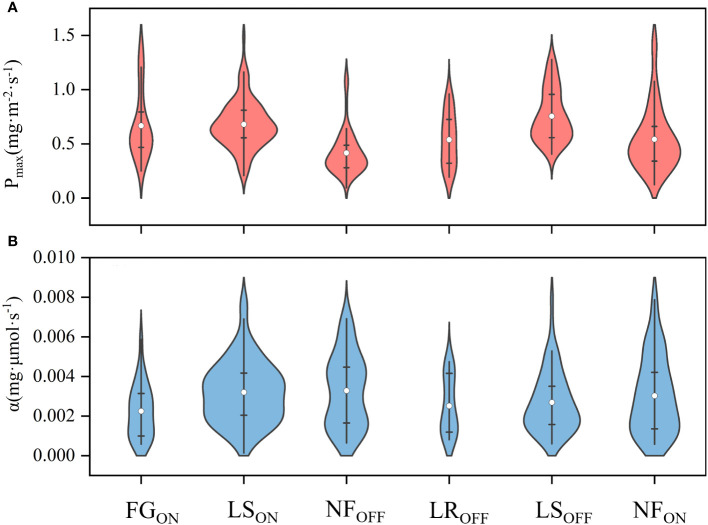
Violin plots of average photosynthetic parameters of P_max_ **(A)** and *α* **(B)** in Moso bamboo forests. White dots are mean values.

As shown in **Figure 6A**, the highest mean P_max_ value was 0.75 ± 0.24 mg·m^-2^·s^-1^ in LS_OFF,_ and the lowest was 0.42 ± 0.20 mg·m^-2^·s^-1^ in NF_ON_, while the highest and lowest values for both on- and off-years corresponded to the LS and NF periods, similar to GEP. As shown in **Figure 6B**, the maximum value of *α* appeared at NF_ON,_ and the minimum value was at NF_ON_. According to the quartiles, the distribution of P_max_ was more concentrated in LS_ON_ and more homogeneous in LR_OFF_. The distribution of *α* is more discrete relative to that of P_max_.

### 3.3 Analysis of the drivers of carbon flux throughout the full phenological cycle

**Figure 7:**
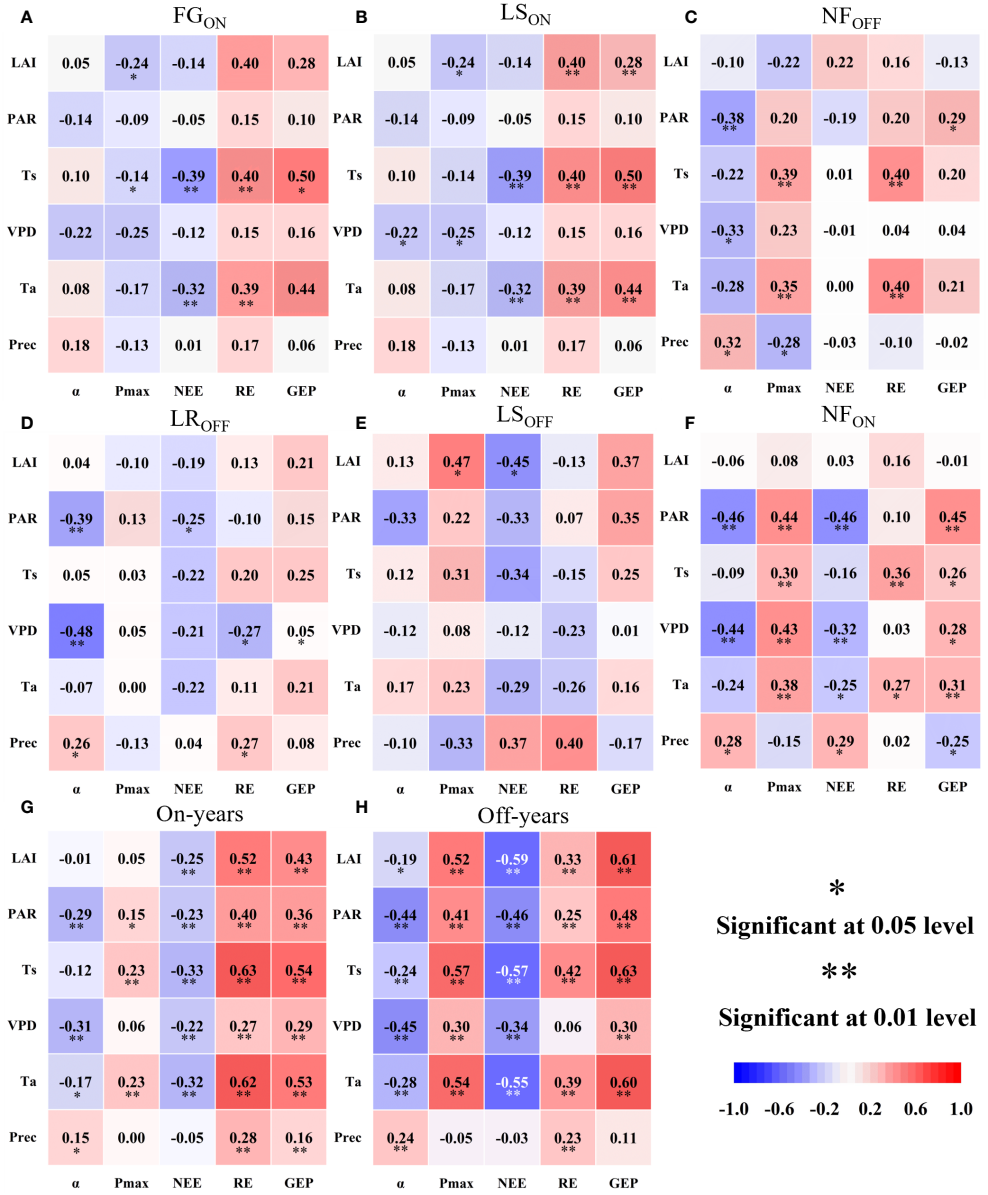
Pearson’s correlation analysis of photosynthetic parameters and carbon fluxes with six biotic and abiotic factors over the full phenological cycle **(A–F)** and on-and off- years **(G, H)** of Moso bamboo forests.

As shown in **Figure 7**, carbon fluxes during the six phenological periods were most closely correlated with temperature, and the correlation with Ts was higher than that with Ta, with the highest correlation in FG_ON_ and the lowest in LR_OFF_ and LS_OFF_. Of the three carbon fluxes, the correlation with temperature varied considerably during the early, middle, and last part of each year. The highest correlation between temperature and NEE was observed in FG_ON_ and LR_OFF_, with Ts and Ta being highly significantly correlated with NEE in the FG_ON_ stage (0.26 and 0.31, *P*< 0.01, respectively) but not in LR_OFF_. The highest correlation with RE was in NF_ON_ (Ts: 0.36), *P*< 0.01; Ta: 0.27, *P*< 0.05) and NF_OFF_ (Ts: 0.40, *P*< 0.01; Ta: 0.39, *P*< 0.01). The highest correlation with GEP was in the LS_ON_ (Ts: -0.42, *P<* 0.05; Ta: -0.32) and LS_OFF_ periods (Ts: 0.25, *P*< 0.05; Ta: 0.21). Moisture factors (Prec and VPD) correlated with carbon fluxes to a lesser extent than temperature, which was significant at LS_OFF_ and NF_ON_ (LS_OFF_, Prec, and VPD to RE: 0.27, *P*< 0.05 and -0.27, *P*< 0.05, respectively; NF_ON_, Prec to NEE and GEP: 0.29, *P*< 0.05 and -0.25, *P*< 0.05, respectively, and VPD to NEE and GEP: -0.32, *P*< 0.01 and 0.28, *P*< 0.05, respectively). Meanwhile, the correlation of moisture factors with carbon fluxes in the early, middle, and last stages of the year showed a different pattern from that of temperature, with the highest correlation being with respiration in all remaining periods except for NF_OFF_, which was the lowest. The correlation between PAR and carbon fluxes was mainly with NEE (negative) and GEP (positive) and was dominated by significant correlations between LS_OFF_ and NF_ON_ (-0.25, *P*< 0.05 for PAR to NEE in LS_OFF_; -0.46, *P*< 0.01 and 0.45 for PAR to NEE and GEP in NF_ON_, *P*< 0.01). The response of carbon fluxes to LAI was mainly in NF_OFF_, with correlations of 0.40 (*P*< 0.01) and 0.28 (*P*< 0.01) with RE and GEP, respectively, and LS_OFF_, with a correlation of -0.45 (*P*< 0.05) with NEE. Interannually, the six factors were significantly correlated with carbon fluxes, with Ts being the most highly significant and Prec the lowest.

For photosynthetic parameters of **Figure 7**, the correlation with temperature was high in the stem growth stage (FG_ON_), with water in the LS, and the effect of PAR was mainly seen after leaf formation (LS, NF). Interannually, the correlation of LAI with photosynthetic parameters was higher in the off-years (LAI to P_max_ and *α* were 0.52, *P*< 0.01 and -0.19, *P*< 0.05, respectively) than that in on-years (LAI to P_max_ and *α* were 0.07 and -0.01, respectively), with the former being significant and the latter not. Meanwhile, similar to carbon fluxes, both had temperature as the most significant driver.

**Figure 8:**
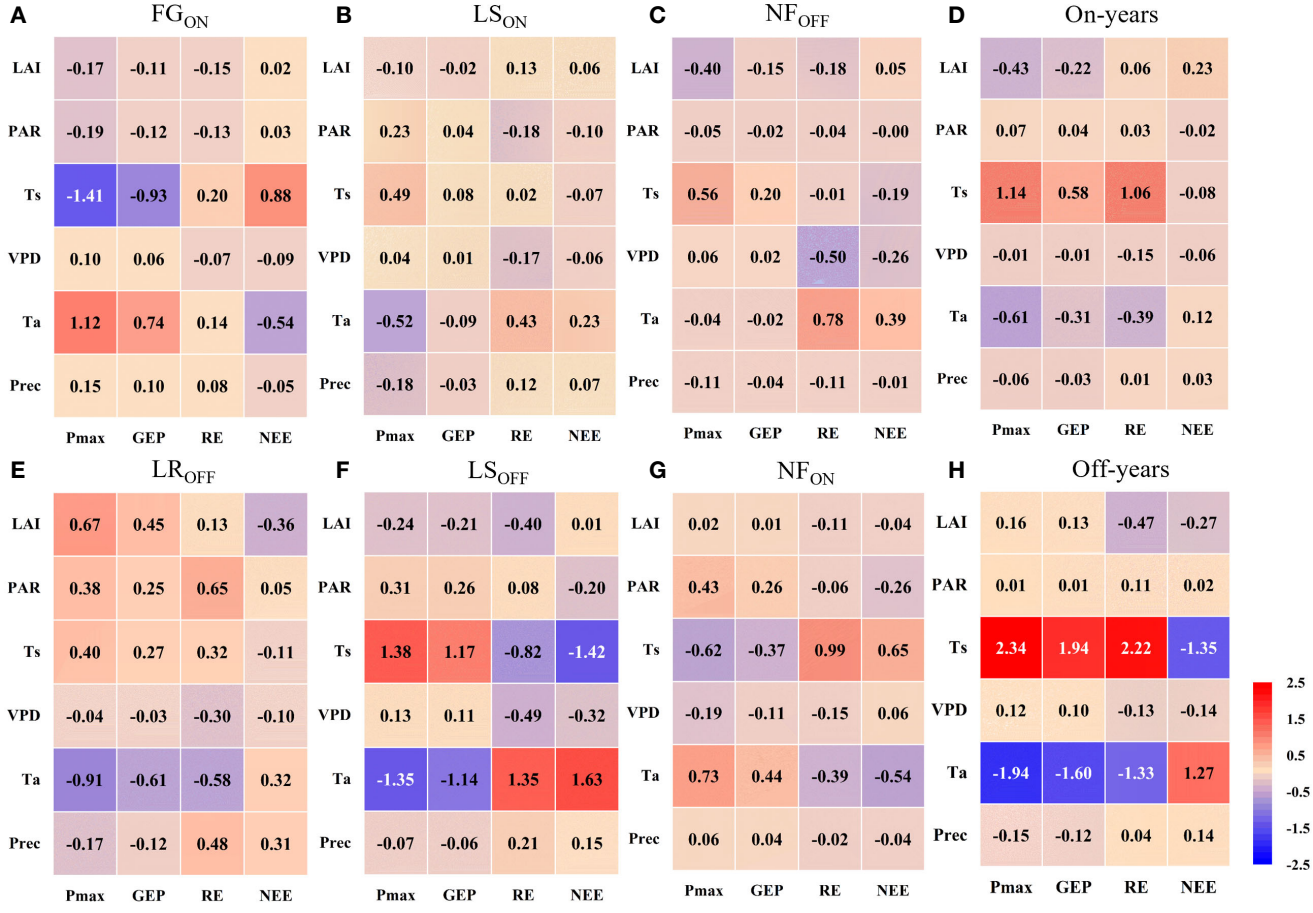
Path coefficients among factors in the full phenological periods **(A–C, E–G)** and on-and off- years **(D, H)** in Moso bamboo forests.

The path analysis results of biotic and abiotic factors on carbon fluxes during different phenological periods are shown in **Figure 8**, and the complete PCs are shown in **Appendix A**. **Figure 8** shows that Ta, Ts, and LAI were the most influential factors on the P_max_ of Moso bamboo, where Ta acted as a facilitator in FG_ON_ and NF_ON_ (PC = 1.12 and 0.73, respectively), and an inhibitor in LR_OFF_, LS_ON_, and LS_OFF_ (PC = -0.91, -0.52, and -1.35, respectively). Ts acted as a facilitator in LS_ON_, LS_OFF_, and NF_OFF_ (PC = 0.49, 1.38, and 0.56, respectively) and an inhibitor in FG_ON_ and NF_ON_ (PC = -1.41 and -0.62). LAI acted as a facilitator and inhibitor in LR_OFF_ (PC = 0.67) and NF_OFF_ (PC = -0.40), respectively. The direct effect of P_max_ on GEP was mainly as a facilitator, with PCs ranging from 0.17 to 0.85 (**Supplementary Figure S1**), whereas the indirect effect of the biotic and abiotic factors on GEP was mainly realized through P_max_, whose effect on P_max_ was similar. On an interannual scale, Ts dominated the increasing effects of P_max_ and GEP in both on- and off-years (mean PC = 3.48 and 1.94, respectively), and Ta acted as a suppressor (mean PC = -1.27 and -0.96, respectively).

Overall, all factors except Prec dominated the carbon sink (NEE reduction) at different stages; FG_ON_, LR_OFF_, LS_ON_, LS_OFF_, NF_ON_, and NF_OFF_ were dominated by Ta (PC = -0.54), LAI (PC=-0.36), PAR (PC = -0.10), Ts (PC = - 1.42), Ta (PC = -0.54), and VPD (PC = -0.26). The dominant factors that contributed to the carbon source (NEE increase) in Moso bamboo forests were Ts and Ta, where Ts was mainly in FG_ON_ (PC = 0.88) and NF_ON_ (PC = 0.65), and Ta was in LR_OFF_ (PC = 0.32), LS_ON_ (PC = 0.23), LS_OFF_ (PC = 1.63), and NF_OFF_ (PC = 0.39). From the interannual results, Ts was overall the most dominant driver of the increase in carbon sinks in both on- and off-years, with a PC of -0.08 in on-years and -1.35 in off-years. The factors that contributed most to the increase in NEE were LAI and Ta in on- and off-years, with PCs of 0.23 and 1.27, respectively.

## 4 Discussion

### 4.1 Carbon fluxes and photosynthetic parameters of full phenological cycle

Although similar environmental elements are present in FG_ON_ and LR_OFF_, the absolute values of NEE and GEP are higher in FG_ON_ than in LR_OFF_, probably due to more carbon fixed in a short period by the “explosive growth” of the on-years (Song et al., 2016). In contrast, the LR_OFF_ stage consisted of leaf replacement during the same period, resulting in an overall lower photosynthetic capacity than that of the former (Gu et al., 2019). Therefore, a significant difference could be seen in carbon fluxes between the two periods. Furthermore, based on the fact that photosynthetic parameters can somewhat reflect the magnitude of photosynthetic capacity (Lin et al., 2022), the average P_max_ of FG_ON_ was not only higher than that of LR_OFF_ but also higher than that of the previous phenological period (NF_OFF_), suggesting that high carbon sequestration rates during the “explosive growth” period may be due to the rapidly increasing photosynthetic capacity (Song et al., 2017), which also contributed to the bimodal NEE trend. We also noticed a decreasing NEE trend in June, which may be due to the high rainfall during the rainy season, which reduces photosynthesis on the one hand and increases soil respiration on the other hand, thus leading to decreased NEE (Chen et al., 2016). Moreover, anthropogenic factors also somewhat affected the carbon flux of the Moso bamboo forest ecosystem, mainly manifested in the lower absolute values of NEE and GEP in LS_ON_ than those in LS_OFF_, primarily due to the decreased LAI caused by the selection and hooking of old bamboo in the current year, which led to decreased photosynthesis (Zheng et al., 2022). The RE of the full phenological cycle of Moso bamboo forests is similar to that of other forests and is also mainly influenced by temperature (Ge et al., 2020). We noted the proximity of RE and LR_OFF_ in FG_ON_ and the high transpiration in both periods (Gu et al., 2019), which may provide evidence for further arguments on the “explosive growth” of new Moso bamboo and the similar amount of nutrients utilized for leaf replacement in old bamboo.

From the photosynthetic pattern of Moso bamboo forests in on- and off-years, the average P_max_ of on-years is higher than that of off-years, and the average GEP is also slightly higher than that of off-years. P_max_ may be an important reason for the difference in the GEP of Moso bamboo forest ecosystems, and simultaneously, the changing pattern of GEP also verifies the previous sample plot scale observation experiment results (Zhang et al., 2011; Xu et al., 2016b). We also observed that P_max_ and *α* negatively correlated in Moso bamboo forest ecosystems, while other subtropical forests usually show positive correlations (Lin et al., 2022; You et al., 2022), the reasons for which need to be further investigated in depth.

### 4.2 Carbon fluxes and photosynthetic parameters in response to biotic and abiotic factors

Based on six factors acting on NEE through P_max_ and through RE (**Appendix A**), we obtained the direct effects of biotic and abiotic factors on Pmax and RE as well as their indirect effects on NEE. Firstly, according to the pathway of “factors - P_max_ – GEP – NEE”. Ta, Ts, and LAI had the most direct impact on P_max_, with Ts dominating in LS_ON_, LS_OFF_, and NF_OFF_ (**Figures 8B, C, F**), Ta dominating in FG_ON_ and NF_ON_ (**Figures 8A, G**), and LAI dominating in LR_OFF_ (**Figure 8E**). NEE pathway showed different indirect impacts by biotic and abiotic factors, in FG_ON_ and LR_OFF_, the factor with the strongest promotion of P_max_ played a dominant role in the increase in sinks (NEE reduction) in Moso bamboo forest ecosystems (**Figures 8A, E**). However, from the pathway of “factors – RE – NEE”. We can see the same factors that dominated RE suppression during the LS_ON_, LS_OFF_, NF_ON_, and NF_OFF_ periods also dominated sink enhancement (NEE increase) in Moso bamboo forest ecosystems (**Figures 8B, C, F, G**). The indirect effects of these two pathways on NEE suggesting that the photosynthetic capacity of Moso bamboo forest ecosystems plays a dominant role in increasing sinks when new bamboo grows explosively and old bamboo changes its leaves. This may be because the most important feature of FG_ON_ and LR_OFF_ lies in leaf change, which overshadows respiration in the change in photosynthetic capacity (Song et al., 2016; Mei et al., 2020) and further explains the important role of LAI in increasing carbon sinks by increasing photosynthesis (Gitelson et al., 2017). Contrastingly, in the remaining four periods, larger respiration was the main cause of lower carbon sinks.

Although RE played a dominant role in changes in NEE, except VPD in off-years, we found significant positive correlations (*P*< 0.01) between RE and the factors under interannual variation (**Figures 7G, H**). It revealed that respiration is overly sensitive to environmental responses, especially temperature factors (Ta and Ts). Meanwhile, respiration during LS periods were subject to a combination of water and heat (**Figures 7B, E**), which may be due to LS periods were the longest stage of six full phenological periods occurring in the in the summer and early autumn, with the presence of extreme climatic factors such as high/low temperatures, droughts and heavy precipitation (Du et al., 2020; Jia et al., 2020).

Both correlation and path analysis showed that temperature had the most important effect on NEE and RE in Moso bamboo forest ecosystems, but Ta and Ts acted in different directions, Ts focuses on the effects on soil, root, and biological respiration in the belowground portion of the body (Tang et al., 2015; Jiang et al., 2016; Ge et al., 2020), whereas Ta focuses on aboveground respiration in the stem and leaf biomass (Wang et al., 2021). In addition, the interannual PC of Ts to RE was higher than that of Ta (**Figures 8D, H**), indicating that the subsurface fraction contributes more to respiration, and thus, reducing soil respiration has the most pronounced effect on sink enhancement (Luo and Zhou, 2010; Oikawa et al., 2017).

Comparatively, water factors (VPD, Prec) and PAR drove carbon fluxes much less than did temperature, with VPD showing a dominant role in respiratory inhibition only during the NF_OFF_ phase (**Figure 8C**), which may indicate that elevated VPD in winter may disrupt plant epidermal stomata (Hsu et al., 2021). PAR is intrinsically unrelated to the maximum photosynthetic capacity of the plant (Lin et al., 2022) and has a relatively low impact on respiration (Liu et al., 2018). However, it regulates GEP primarily by affecting real-time photosynthesis in Moso bamboo forests (Xu et al., 2016b).

Both correlation and path analysis showed that the six biotic and abiotic factors selected in the current study strongly correlated with carbon fluxes changes in Moso bamboo forests (**Figures 7**, **8**). However, the correlations and the mechanisms of direct and indirect effects of six factors on photosynthetic parameters as well as carbon fluxes varied across phenological periods. For example, the correlation between LAI and P_max_ were significant negative in certain periods (e.g., FG_ON_, LS_ON_; **Figures 7A, B**), but were significant positive in off-years (**Figure 7H**). On the one hand, it suggested that the greater LAI represents stronger photosynthesis in Moso bamboo forests (Lin et al., 2022; You et al., 2022). On the other hand, the negative direct relationship may be caused by the uncertainties of large-scale remote sense monitoring capture changes at the site scale (Tian et al., 2002) when a rapid leaf spreading during FG_ON_ period. Moreover, the single-peaked shape of LAI time series differs with the bimodal of P_max_ in on-years (**Figure 3**) also indicate that there may have a certain delay of MODIS LAI product at site scale implementation (Heiskanen et al., 2012), despite we have applied particle filter algorithm to improve its accuracy. Therefore, analyzing the abiotic response mechanisms of carbon fluxes in Moso bamboo forests in the full phenological cycle is particularly important (Song et al., 2017; Chen et al., 2018; Zhou et al., 2019).

### 4.3 Uncertainty analysis

In this study, we used EddyPro to process 10 Hz of raw flux data to a 30-min time scale and then culled and interpolated it to form a complete flux time series based on standard FLUXNET and ChinaFLUX processing methods, although the processing was subject to some uncertainties (Kim et al., 2020; Zhu et al., 2022). On the one hand, the choice of friction velocity threshold is among the most important causes of uncertainty in rejecting anomalous data from carbon flux observations (Pastorello et al., 2020). Its value generally varies with forest type, and the observed stand conditions of the friction velocity threshold are inconsistent, generally ranging from 0.2 to 0.35 m·s^-1^ (Xu et al., 2016a; Liu et al., 2018; Zhou et al., 2019). Therefore, we adopted 0.2 m·s^-1^ as the threshold based on the variation characteristics of the observed data with some scientific basis. On the other hand, mean diurnal variation method is among the common data interpolation methods (Moffat et al., 2007). However, interpolation of long-missing observations increases the uncertainty of the results due to changes in environmental factors (Richardson et al., 2007). In addition to the mean diurnal variation method, look-up table and ANNs have been relatively hot during the recent years (Mahabbati et al., 2021). However, on the one hand, the performance of look-up table in gap-filling of extralong gaps is not well known (Kim et al., 2020), on the other hand, despite their reliable performance, ANNs – and generally all other machine learning algorithms – face some challenges. Over-fitting, for instance, is a big concern and can happen when the number of degrees of freedom is high, while the training window is not long enough or the quality of the training dataset is low (Zhu et al.,2022). In the present study, although mean diurnal variation method was used, we interpolated day- and nighttime data separately, and flexibly set the fitting window according to meteorological conditions (Xu et al., 2016a), improving data accuracy to some extent.

Carbon fluxes in forest ecosystems of different types and regions does not respond uniformly to biotic and abiotic factors (Baldocchi et al., 2018; Zhao et al., 2022). For example, precipitation is the main driver of carbon flux in African ecosystems (Merbold et al., 2009), whereas the NEP of Dahurian larch forest ecosystems in northeast China is mainly dominated by VPD (Wang et al., 2008). Moreover, three Canadian boreal black spruce forests differed in their patterns of response to light and temperature (Bergeron et al., 2007). For Moso bamboo forest ecosystems, previous studies indicated that Ta is the most influential factor of RE on a monthly scale, and PAR, Ts, and VPD influence NEE the most (Liu et al., 2018; Chen et al., 2019). Therefore, although all common biotic and abiotic factors were not included in the current study, the selected factors were those significantly affecting carbon fluxes in Moso bamboo forest ecosystems, giving reliable results.

Pathway modeling is crucial in solving the PCs, and different models can lead to different results (Klem, 1995). For example, Wetzels et al. (2009) discussed the different between SEM and PLS pathways, and Papin et al. (2004) examined the differences in the results of path analysis conducted using basic and extreme pathways. In the present study, the overall pathway frameworks (**Figure 2**) of “environmental factors - photosynthetic parameter – GEP - NEE” and “environmental factors – RE - NEE” were developed based on the importance of photosynthetic parameters in the carbon cycle (Lin et al., 2022) and the significant influence of environmental factors on respiration (Li et al., 2020). However, time and labor cost constraints led to the insufficient consideration of biotic factors (e.g., chlorophyll fluorescence, LAI, etc.) acquired by remote sensing in this modeling framework, which will be improved in future studies.

## 5 Conclusion

In this study, based on flux observations and micrometeorological observations using the eddy covariance technique and field observation experiments, we analyzed the changes in carbon fluxes and photosynthetic parameters of Moso bamboo forest ecosystems in the full phenological cycle of 2011–2015. We further analyzed their direct and indirect drivers using correlation and path analyses. The main conclusions were as follows:

1. The photosynthetic capacity of the full phenological cycle was the strongest in LS, with an average P_max_ of 0.75 and 0.68 mg·m^-2^·s^-1^ for LS_ON_ and LS_OFF_, respectively. NF_OFF_ had the weakest photosynthetic capacity, with an average P_max_ of 0.42 mg·m^-2^·s^-1^. The carbon sink and photosynthetic capacities of the ecosystem were synchronous, and the top two are LS_OFF_ and LS_ON_, whose average NEEs were -23.85 and -19.04, respectively. NF_OFF_ showed the weakest capacity with the value of -3.37 gC·m^-2^. Interannually, photosynthetic capacity was higher in on- than in off-years, with a mean P_max_ of 0.63 and 0.57 mg·m^-2^·s^-1^, respectively, and mean NEE of -1181.14 and -948.27 gC·m^-2^·a^-1^, respectively.
2. Ts was the most important driver of the effects of abiotic factors on the annual photosynthetic and carbon sequestration capacities of the Moso bamboo forest ecosystems, especially during bamboo forests focused on stem and underground part growth showcasing direct effects on Pmax (NF_ON_: -0.62, FG_ON_: -1.41) and corresponding indirect effects on NEE (NF_ON_: 0.65, FG_ON_: 0.88). In contrast, when the focus shifted to leaf growth, Ta emerged as the main driver, with PCs of Ta concerning Pmax being -0.91, -0.52, and -1.35 for LR_OFF_, LS_ON_, and LS_OFF_, respectively, and indirect effects on NEE of 0.32, 0.23, and -1.42, respectively.
3. The increase in LAI significantly enhanced the net carbon capacity, especially in off-years, exhibiting the highest correlation with NEE was the among the six factors (-0.59). The effect of LAI on P_max_ and NEE had a hysteresis during the six phenological periods when leaves were still growing, and LAI promoted P_max_ when leaf growth was stable in the long period, e.g., its PCs with P_max_ were 0.67, 0.02, and 0.16 in LR_OFF_, NF_ON_, and off-years, respectively.

## Data availability statement

The original contributions presented in the study are included in the article/**Supplementary Material**, further inquiries can be directed to the corresponding author/s.

## Author contributions

CX: Conceptualization, Data curation, Investigation, Methodology, Software, Writing – original draft, Writing – review & editing. FM: Methodology, Supervision, Writing – review & editing. HD: Methodology, Supervision, Writing – review & editing. XL: Data curation, Supervision, Writing – review & editing. JS: Data curation, Software, Writing – review & editing. FY: Data curation, Software, Writing – review & editing. XT: Data curation, Writing – review & editing. ZZ: Data curation, Writing – review & editing. NY: Data curation, Writing – review & editing.
