# Supplementary figures and images for: Full phenology cycle carbon flux dynamics and driving mechanism of Moso bamboo forest
Source: Front Plant Sci. 2024 Feb 26;15:1359265. doi: 10.3389/fpls.2024.1359265 (PMC10935103; doi:10.3389/fpls.2024.1359265)

# Appendix A. Supplementary materials


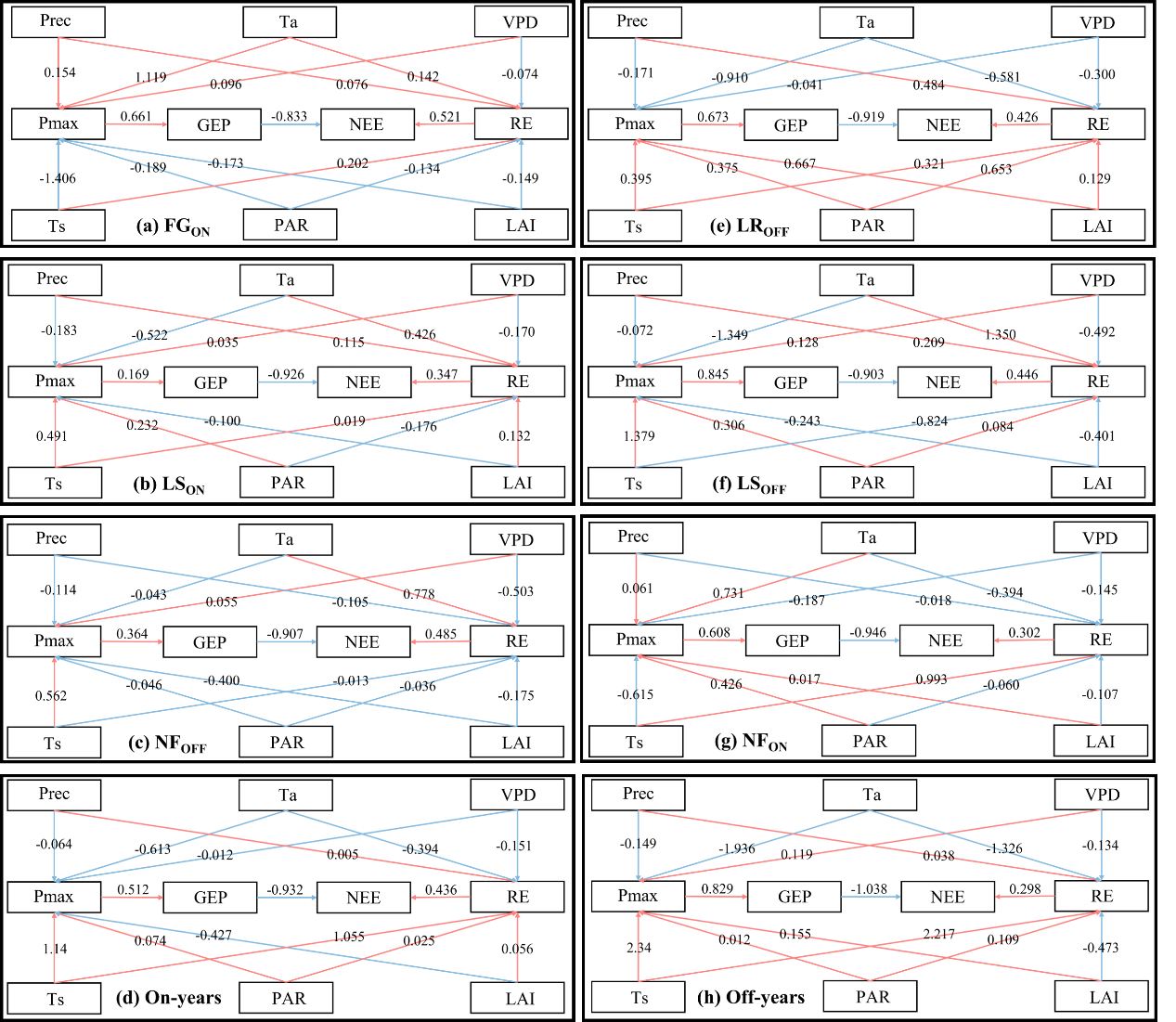


**Fig. S1**. Pathway model of the full phenological cycle

Supplement: Supplementary file 1 [file DataSheet_1.docx]
